# Rush Medical College Commencement

**Published:** 1855-03

**Authors:** 


*Rush Medical College Commencement.*

The eleventh annual College term closed on Wednesday the 21st of February, and the exercises of the public commencement took place in Metropolitan Hall, on the evening of that day. The degree of Doctor of Medicine, was confered on 41 candidates, after which a valedictory address was delivered by Prof. D. Brainard. The exercises were interesting and witnessed by a large audience— The past college term has been a gratifying one in several respects. While, as far as we can learn, the number of students attending the medical schools throughout the country has considerably diminished, our class has maintained its full number; and what is still better, a very large proportion of them exhibit evidence, not only of medical attainments, but also of a much more satisfactory knowledge of general science and literature.

The following are the names of the graduates, with the titles to their inaugural theses, viz:

| | | |
|---|---|---|
| Christopher Goodbrake, | Ills. | on Diagnosis. |
| Homer C. Rawson, | O. | ,, Cholera. |
| Thomas B. Hanna. | Ills. | ,, Opium. |
| Horace C. Clapp, | Mich. | ,, Disease. |
| Leroy H. Kennedy, | Ind. | ,, Scarlatina. |
| Freeborn F. Hoyt, | Min. Ter. | ,, Gonorrhoea. |
| George T. Goldsberry, | O. | ,, Incontinence of Urine. |
| William Van Nuys, | Ind. | ,, Mercury. |
| James Ford, | ,, | ,, Hard Engorgements of the Uterus. |

| | | |
|---|---|---|
| Martin Wiley, | Ills. | ,, the Study of Medicine. |
| John McHugh, | ,, | ,, Typhoid Fever. |
| Henry Van Meter | ,, | ,, the Influence of the Mind on Disease. |
| Elias Wenger, | ,, | ,, Management of Parturition. |
| Ross W. Pierce, | Ind. | ,, the Digestive Function. |
| Michael R. Chadwick, | ,, | ,, the Vis Medicatrix Naturæ. |
| Thaddeus M. Crombie, | Ills. | ,, Syphilis. |
| Mordecai Davis, | ,, | ,, the Requisites of the Physician. |
| Berry W. Cooper, | Ind. | ,, Typhoid Fever of Madison Co., Ind. |
| Isaac Rice, | Ills. | ,, Allopathy. |
| Hugh Russell, | Wis. | ,, Pneumonia. |
| Charles W. Jenks, | Ills. | ,, Secondary Assimilation. |
| Alonzo L. Hutchinson, | Mich. | ,, the Toxicology of Arsenious Acid. |
| John F. McCarthy, | Ind. | ,, the Mind and its relations to the Body. |
| John W. Trabue, | Ills. | ,, Dysentery. |
| Hiram L. Coon, | Wis. | ,, Digestion. |
| Hiram J. Van Winkle | Ills. | ,, Physicians and their Duties. |
| Elisha G. Horton, | Wis. | ,, Hemorrhage. |
| Soloman S. Clark, | ,, | ,, Physical Diagnosis. |
| Vernon Gould, | Ind. | ,, Semiology. |
| William H. Heller, | Ills. | ,, Scarlatina. |
| Jason N. Conley, | Ind. | ,, Chloride of Sodium. |
| James Evans, | ,, | ,, Uterine Hemorrhage. |
| Darwin Du Bois, | Ills. | ,, the Physiology and Pathology of the Portal System. |
| Lewis C. Bicknell, | Wis. | ,, Pneumonia. |
| Charles Gorham, | Ills. | ,, Milk-Sickness. |
| James C. McMurtry, | ,, | ,, Hysteria. |
| James F. Grove, | N.Y. | ,, Diagnosis. |
| Jesse Barbre, | Ills. | ,, Typhoid Fever. |
| James M. Sudduth, | ,, | ,, Fevers. |
| Allen A. Rawson, | O. | ,, the Lancet. |
| George A. Byrns, | Ills. | ,, Acute Rheumatism. |

In addition to the above, an excellent Thesis, written in Latin, was received from Dr. E. S. Cooper of Henderson, Ills., on whom the College had previously conferred the honorary degree of M. D.

"*Our Easy Chair*"

Under this caption, our friend Prof. A. B. Palmer, one of the Editors of the Peninsular Journal of Medicine, published at Ann Arbor, Mich., gave, in his January number, a glowing description of a new black-walnut, spring-cushioned, hair-cloth covered, *easy* chair; with writing stand and apparatus attached. So comfortable did the Doctor feel in his new chair, that he thought it conferring a great favor on his readers to inform them that others just like his could be obtained by sending an order with *eleven* dollars enclosed, to Hewson and Parsons of Auburn, N. Y. Now in this matter of chairs we are a little ahead of our friend Palmer. For on going to the office a few evenings before the close of the recent college term, we were met by a committee of members of the college class and presented with a chair, that *is a chair*.

It is not made of "black-walnut," nor covered with "hair-cloth;" neither was it imported from the "great State Industrial Institution at Auburn," *alias* the State Prison, but its frame work is of the best curl-maple, the cushions of the arms, back, and seat of the richest velvet, the latter resting on easy springs, and the whole put together in the most elegant style by *honest* men of Chicago.—With the chair was presented, not a writing leaf and drawer, but what was a great deal better, viz: one of the most valuable *gold pens and pencil* we ever saw.

And then the kind words that accompanied their presentation by H. C. Clapp, M. D., the representative of the committee; how pleasant, how cheering were they?—We could only give in return for all this, a pledge never to *sleep in the easy chair* while science and humanity called for action, nor to use the *golden pen* for any other purpose than the upbuilding of the profession of our choice.

*Foreign Honors*.—We were pleased to learn, a few days since, that our Colleague, Prof. Daniel Brainard, had been elected corresponding member of the Medical Society of Geneva, in Switzerland.

*History of the American Medical Association.*

Little more than one year since, the enterprising publisher of the New-Jersy Medical and Surgical Reporter, commenced publishing a series of articles on the History of the American Med. Association, accompanied by portraits and Biographical sketches of the presidents of that body. The articles have now reached seven in number, and present a very interesting history of the origin and progress of the Association up to 1851. The portraits thus far given are those of Drs. Nathaniel Chapman, Alexander H. Stevens, John C. Warren, Reuben D. Muzzy, James Moultrie, Beverly B. Welford, and Jonathan Knight. These are all excellent plates, and are alone worth five times the price of the Journal in which they appear. We trust that the same enterprize which has called forth this history and the accompanying portraits will give both to the public in a separate volume, as soon as the series is completed.

---

*Index to Volume 3d.* We had entirely forgotten to prepare an index and title page for Volume 3d of the new series, until reminded of it by a Subscriber—The defect shall be supplied in our next issue—Several have also applied for back numbers to make their volumes complete. These shall be answered during the coming week.

